# The tumor as an organ: comprehensive spatial and temporal modeling of the tumor and its microenvironment

**DOI:** 10.1186/s12859-016-1168-5

**Published:** 2016-08-24

**Authors:** Naamah Bloch, David Harel

**Affiliations:** Department of Computer Science and Applied Mathematics, Weizmann Institute of Science, 234 Herzl st, 7610001 Rehovot, Israel

**Keywords:** Computational models, Biological systems, Statecharts, Tumor and its microenvironment, Visualization

## Abstract

**Background:**

Research related to cancer is vast, and continues in earnest in many directions. Due to the complexity of cancer, a better understanding of tumor growth dynamics can be gleaned from a dynamic computational model. We present a comprehensive, fully executable, spatial and temporal 3D computational model of the development of a cancerous tumor together with its environment.

**Results:**

The model was created using Statecharts, which were then connected to an interactive animation front-end that we developed especially for this work, making it possible to visualize on the fly the on-going events of the system’s execution, as well as the effect of various input parameters. We were thus able to gain a better understanding of, e.g., how different amounts or thresholds of oxygen and VEGF (vascular endothelial growth factor) affect the progression of the tumor. We found that the tumor has a critical turning point, where it either dies or recovers. If minimum conditions are met at that time, it eventually develops into a full, active, growing tumor, regardless of the actual amount; otherwise it dies.

**Conclusions:**

This brings us to the conclusion that the tumor is in fact a very robust system: changing initial values of VEGF and oxygen can increase the time it takes to become fully developed, but will not necessarily completely eliminate it.

**Electronic supplementary material:**

The online version of this article (doi:10.1186/s12859-016-1168-5) contains supplementary material, which is available to authorized users.

## Background

Cancer, a state of abnormal growth and regulation of cells, which proliferate in an uncontrolled way, is among the leading causes of mortality worldwide. Researching cancer is of high importance.

The dynamic microenvironment in which a tumor originates, also termed the *stroma*, plays a critical role in tumor initiation and progression, and may be an important factor in developing therapeutic approaches. For years, research has focused on understanding the transformation of normal cells into neoplastic or cancerous ones. However, it has become evident that the surrounding environment of the tumor cells is equally important. Cells that surround the tumor and can take part in its development include innate and adaptive immune cells, fibroblasts [1], cells that line the blood and lymphatic vessels, and the proteins that make up the structural component–the *extra cellular matrix* (ECM). The tumor cannot survive or progress on its own [2, 3]; it entirely depends on this dynamic microenvironment in which it originates and the bi-directional interactions with this surroundings [4, 5]. These include secretion of signals or cell-cell interactions and can act to either enhance or block tumor formation. The conditions within the tumor’s microenvironment differ considerably from those in normal tissue.

Angiogenesis, the process of new blood vessels growing from pre-existing ones, is an essential step in the transition of tumors from a dormant state to a malignant one. The new blood vessels grow towards the tumor and feed it with large supplies of oxygen and nutrients. Without angiogenesis, tumors cannot grow beyond the size of 1 mm^3^. The process leading to angiogenesis begins when the tumor cells lack oxygen (a state of hypoxia), and is followed by a series of events, orchestrated by a variety of activators and inhibitors.

There is a balance between the pro- and anti-angiogenic factors and when this balance tips in favor of the pro-angiogenesis, the onset of angiogenesis, or the angiogenic switch, is induced [6]. The tumor cells release (or cause nearby cells to produce) angiogenic factors that stimulate the formation of blood vessels and recruit them to the tumor’s area [7].

A major activator of the proliferation and migration of the endothelial-cell (the vessels lining) is the *vascular endothelial growth factor* (VEGF). The endothelial cell at the tip of the emerging vessel starts to migrate towards the angiogenic signals by sensing the concentration gradient [8, 9], while in the stem of the newly formed vessel the endothelial cells proliferate, adhere to each other and create a lumen (the inner space of the tubular vessel). Indeed, blocking angiogenesis by inserting anti-angiogenic factors has been a strategy for clinicians in their efforts to arrest tumor growth [10].

Computational approaches have become a big part of biological research. Computational modeling of biological systems provides a means to integrate a large amount of data and generate a comprehensive overview of the behavior of a system as a whole [11, 12]. Furthermore, being able to visualize animations of the model in operation can significantly aid in gaining a clear understanding of complex biological behavior, and together can serve as a beneficial way to analyze the system and make new discoveries.

Due to the complexity of cancer, a better understanding of tumor growth dynamics and insights into the cancer’s behavior can be gleaned from a dynamic computational visual model. We have therefore focused on modeling the cancerous tumor and its microenvironment and bringing it to life with an interactive animation tool.

Cancer, given its clinical importance, has been studied in detail, and is continuously under intensive investigation. A multitude of experimental, clinical and theoretical studies exist and have shed light on many aspects of cancer on all levels: the sub-cellular scale (e.g., DNA and proteins) [13, 14], cellular scale (activation, proliferation, interactions) [15, 16], and system scale (cell migration, diffusion, metastasis) [17, 18]. Given its complexity and multiscale nature, a better understanding of tumor growth dynamics can be expected from a suitable approach to computational modeling [19–21].

Extensive attempts have been carried out to model and analyze cancer, or particular facets thereof [22, 23]; for the most part, this is done by traditional mathematical modeling [24–30], using a top-down approach, whereby the behavior of the system is inserted into the model. [31–41]. Modeling work on cancer using agent based methods has also been used ([42, 43]).

Our goal was to create a comprehensive model of the entire system, whereby we first model and only then ask the questions and not the other way around. This approach, sometimes termed ‘executable biology’ [44–49], focuses on designing executable models that mimic complex biological phenomena. It is carried out in a bottom-up fashion, whereby the behavior of each of the elements of the system (e.g., cells) is described individually, and the system’s overall behavior emerges from those of its many elements. The main language used to build our model is the visual formalism of Statecharts.

We connected our Statechart model to SimuLife, an animation tool that we built especially in our group for viewing the behaviors of such biological models, and which thus serves as a sort of front end to them [50]. SimuLife, based on the technique of reactive animation (RA) [51, 52], is 3D, web-based, and easy to use via an intuitive interface. Visualization of the cancer model is important, as it enables one to see the development and morphology of the tumor and its surroundings based on its individual components. It can be used to fine-tune the model, visualize the effect of changing elements or parameter values, and to verify the behavior of the system.

## Results and discussion

We created a comprehensive, fully executable, spatial and temporal 3D computational model that demonstrates the behavior of a typical cancerous solid tumor together with its microenvironment, treating it somewhat like a developing organ.

The model captures the ongoing bidirectional cross talk between the tumor and its surroundings, which plays a critical role in tumor initiation and progression, so that researching the dynamic behavior and morphology of this system via such a model should be of great interest.

The way we chose to build the model is in a bottom-up fashion, whereby the behavior of each of the system’s elements is described individually, using only the fundamental building blocks of that element. The system’s overall behavior emerges from that of the elements thereof, which is the essence of realistic modeling.

### The tumor model in Statecharts

**Fig. 1:**
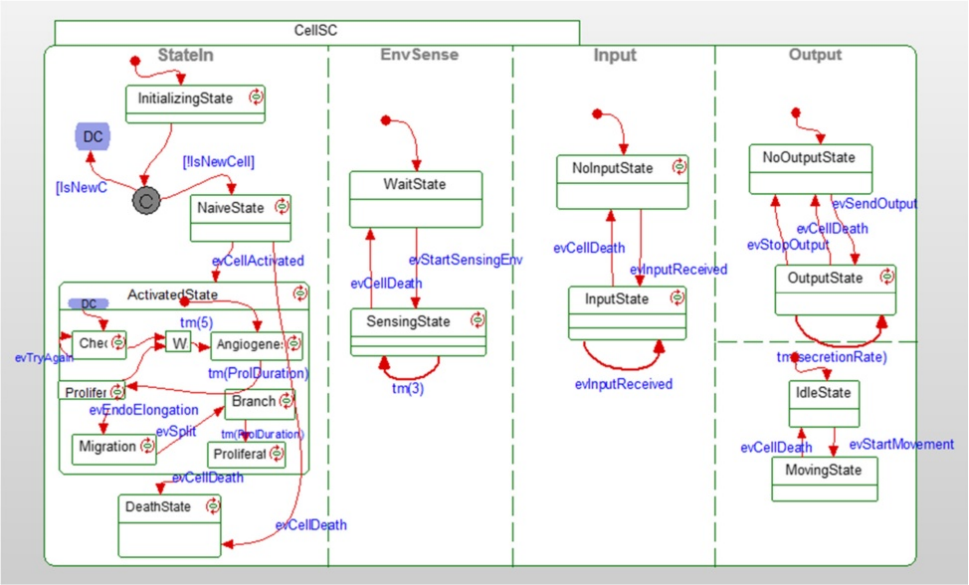
Statechart of a cell. An example of a statechart that was used in the model

Using Statecharts, a generic program of behavior was created for each of the different types of the objects. During an execution of the model many instances of the objects are generated to represent each specific instance taking on its explicit states accordingly (see Fig. 1 for an example of a statechart). This resulted in a comprehensive and reactive computational model.

The tumor and its microenvironment act together as an organ, which defines the ‘world’ of the model. The model begins with a single cancerous cell, which proliferates to gradually form a primary tumor, consisting of cancerous cells that originated from the initial one. Each cell has its specific 3D position within this world and takes on its own behavior, depending on its current state and surroundings.

Controlled by the Statechart driving its behavior, the tumor cell constantly senses its immediate surroundings, and consumes available oxygen at a certain level. If it cannot consume a sufficient amount of oxygen, it will not be able to proliferate, will go into a state of hypoxia and will secrete angiogenic factors (VEGF). The VEGF is secreted in pulses, and continues to be secreted until the cell is able to consume enough oxygen so as not to be in a state of hypoxia. The VEGF diffuses from the specific tumor cell and moves in a random manner. If the cell continues to lack oxygen it enters a state of anoxia, becoming necrotic and no longer active.

A number of blood vessels, made up of individual endothelial cells, are initially located either around the border of the defined world, or at random positions therein. The endothelial cells are of roughly the same size as the tumor cells, and similarly sense their environment constantly. Once they bind to an amount of VEGF above a specified threshold within a certain amount of time, they become activated and begin the process of angiogenesis, where the vessels elongate in a direction that follows the VEGF gradient. This occurs by proliferation of those endothelial cells that have become activated and continues for as long as the endothelial cell continues to bind enough VEGF for its continuation. If the endothelial cell encounters another endothelial cell, it will join it and stop elongating. Activated endothelial cells can also split and branch out of their main vessel if they bind a high amount of VEGF in a short amount of time. Due to the delta-notch inhibition between adjacent cells, once an endothelial cell is activated, the neighboring cells cannot become activated too [53, 54]. The endothelial cells secrete oxygen at a constant rate, and like the VEGF molecule, the oxygen diffuses out of the cell in a random manner and is eliminated when consumed.

In this way, the newly produced blood vessels make their way to the tumor, in the process forming a unique spatial organization, and as a result the tumor continues to grow. Newly produced endothelial cells that do not continue to receive a minimum amount of VEGF cannot survive and eventually cause the death of the vessel of which they are part.

Fibroblast cells [1], which are the main components of the ECM, are initially placed randomly around the tumor. During the model’s execution, those that are close to the tumor cells and have enough oxygen have a greater chance of becoming activated into CAFs (cancer associated fibroblasts) [55]. CAFs secrete VEGF in correlation with their hypoxia state [56], which helps recruit the blood vessels. They also secrete HGF (Hepatocyte Growth Factor), which helps the tumor cells proliferate, as well as degrade the ECM around them, which, in turn, helps tumor cells move. CAFs themselves are motile and move towards the tumor [57, 58] by following the gradient of FGF (Fibroblast Growth Factor) that is secreted by the tumor cells. This type of movement can casue entry of CAFs into the tumor [55].

### The SimuLife animation tool

Whilst the Statechart model holds the information of each of the individual objects, SimuLife makes it possible to visualize the information of all the objects together at once [50]. This helps greatly when developing the model; to be able to see the dynamics of the whole system, verify the behavior, fine-tune the model as a consequence, and examine the effect of the parameters on the system.

### Results analysis

Many parameters were incorporated into in the model. Since we do not use every biological aspect whilst modeling, the parameter values do not represent exact measurement values but only values that are logical relative to each other. In addition, the values of the parameters in the model are unit-less (for a list of parameters, see Additional file 1). What guides us when adjusting parameter values is the behavior of the element in the model and that this matches the actual behavior of the real biological element it represents.

In order to check the correctness of the whole model and understand the behavior of the system, analysis was performed on the data retrieved from the model, and it was compared to existing biological data.

**Fig. 2:**
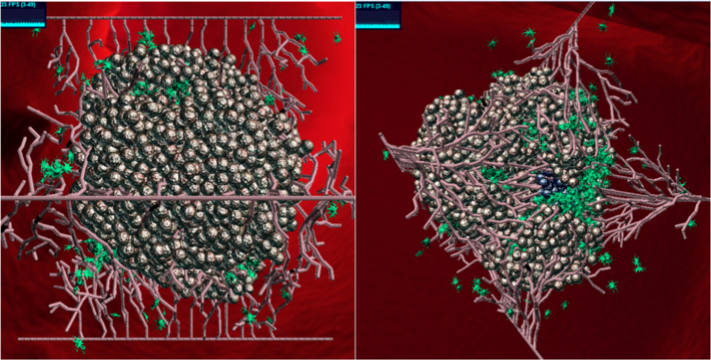
SimuLife image of the tumor. A SimuLife image of the tumor surrounded by vessels and fibroblasts (green) as created by the Statecharts model. Shown from two different angles
  In addition, a necrotic core is formed in the inner part of the tumor where the cells do not receive enough oxygen and undergo necrosis [64, 65] (Figs. 3 and 4). This occurs as the tumor expands and the inner cells are no longer exposed to oxygen. The necrotic core occupies from a few percent of the tumor up to the majority of its volume, depending on the amount of available oxygen.

First, we wanted to verify that the behavior of the system modeled is consistent with that of a real cancerous tumor. This was done by looking at a number of points of comparison:

1. The overall fundamental behavior of a solid tumor, where the malignant cells proliferate into a primary tumor until they lack sufficient oxygen and nutrients, and then send out VEGF in order to recruit new blood vessels, which arrives at the endothelial cells of the original blood vessels [59, 60]. The VEGF molecules will have an affect only after enough molecules have arrived to initiate angiogenesis, a process that can take up to a few days. The vessels, in turn, grow towards the concentration gradient and supply the tumor with oxygen/nutrients, enabling it to continue growing. The vessels arrive at the tumor within anything from a few days to a few weeks [61], creating a new network [6, 10].
  This behavior is found in the model, and when visualized by using Simulife (Fig. 2) compares favorably to images found in [62, 63].

**Fig. 3:**
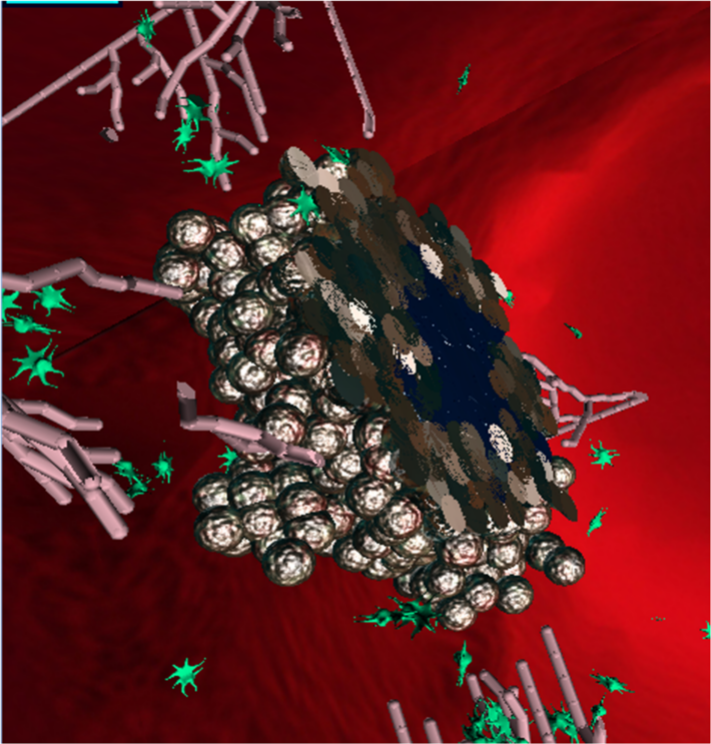
Slicing of the tumor in SimuLife; the necrotic core (dark blue) can be seen

**Fig. 4:**
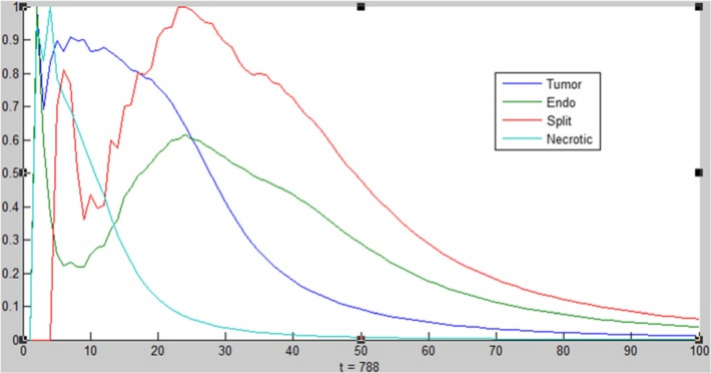
Distribution of cells. Distribution of the different cells as a function of the distance from the center of the tumor (x = 0) at time step 788. Necrotic cells (*light blue*) occur at the center of the tumor. Vessel splits (*red*) occur more as they approach the tumor (*blue*)
  The blood vessels branch more as they approach the tumor (the brush-border effect, [66–68]). This occurs as endothelial cells that are closer to the tumor sense more VEGF and are therefore activated more easily to sprout and form new vessels (Fig. 4).
  The diffusion behavior of the VEGF and oxygen molecules can be verified by their distribution over time. Molecules diffuse in space, forming a symmetric, decreasing gradient [69]. In the model, oxygen is initially present everywhere but is gradually consumed by the tumor and in parallel is secreted by vessels. VEGF is secreted by the tumor (and partly by fibroblasts) and spreads out while being consumed by the endothelial cells (Fig. 5). The molecules diffuse by moving in a random fashion from their point of secretion, gradually occupying available space.

**Fig. 5:**
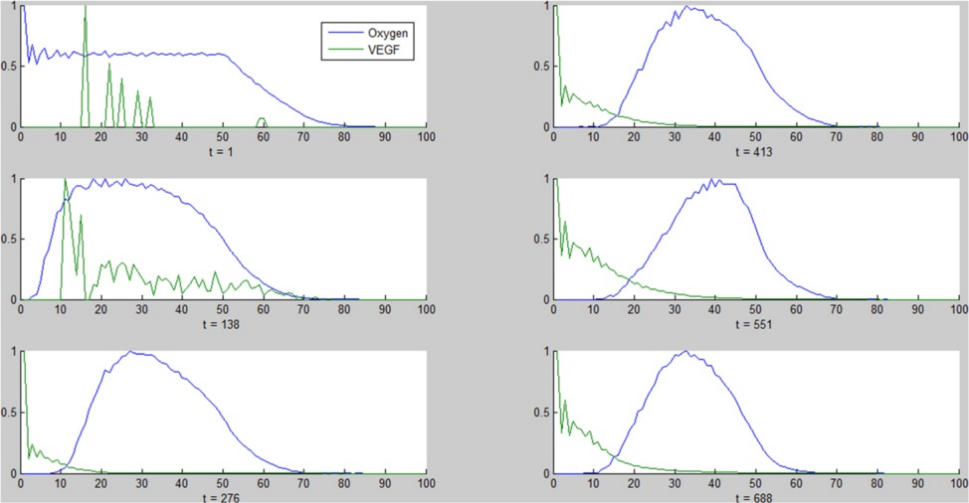
Distribution of molecules. Distribution of oxygen (*blue*) and VEGF molecules (*green*) as a function of the distance from the center of the tumor (x = 0) at different time steps throughout the simulation (left column followed by right column, from top to bottom). Oxygen is initially present everywhere but is gradually consumed by the tumor and in parallel secreted by vessels. VEGF is secreted by the tumor and spreads out while being consumed by the endothelial cells. The VEGF in the first two graphs is due to secretion by fibroblasts and not by the tumor
  It is important to note that the above anticipated behaviors of the system (necrotic core, increased branching near the tumor, and distribution of diffused molecules) emerged bottom-up from the behavior of the individual components and rules of the system, and not by inserting such behavior into the model explicitly.
  2) The dynamic behavior of the various cells and molecules as observed from running and analyzing the model, agrees with that found in literature. In (Fig. 6) we see that the emergent behavior of the system results in an initial linear growth of tumor cells, a halt in the growth (due to lack of oxygen), then growth of endothelial cells, and consequently an exponential growth in the number of tumor cells. This can be compared to results found in [70–73], where the growth of tumor cells and blood vessels exhibit a similar pattern.

**Fig. 6:**
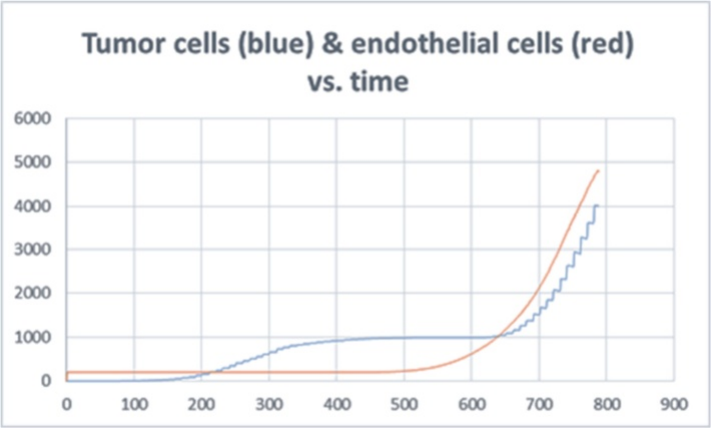
Tumor cell and endothelial cell dynamics in the simulation. The behavior of the tumor cells and vessels in the model can be compared to biological results
2. Further verification included checking that “normal” cases occur in the model. This included testing the system under the following sets of circumstances:
  - No angiogenesis, which resulted in a primary tumor that stopped growing at some point due to low oxygen/nutrient supply, and then started to die off [74–77] (Fig. 7).

**Fig. 7:**
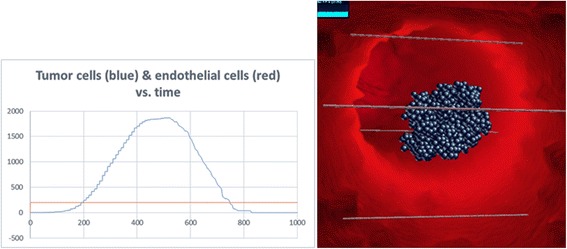
No angiogenesis. Tumor does not develop. Left: tumor cell and endothelial cell dynamics. Right: image of the same run in SimuLife (blue cells are necrotic)
  - Non-cancerous and non-proliferating cells, which are able to live on normally without angiogenesis (Fig. 8).

**Fig. 8:**
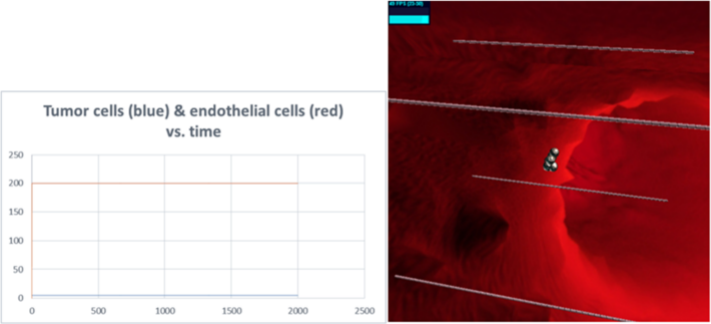
No angiogenesis. Non-cancerous and non-proliferating cells live on. Left: tumor cell and endothelial cell dynamics. Right: image of the same run in SimuLife
  - Blood vessels placed far from the tumor cells (at ~300 μm instead of ~200 μm, where each position in the model corresponds to 5 μm), which results in no angiogenesis, and hence not enough oxygen, leading to a non-active tumor [77–79] (Fig. 9).

**Fig. 9:**
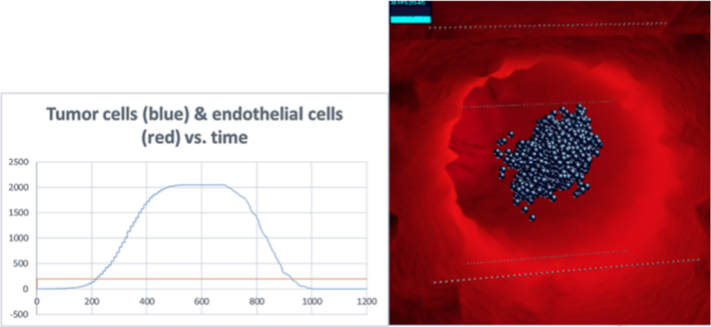
Initial blood vessels are too far away. Tumor does not develop. Left: tumor cell and endothelial cell dynamics. Right: image of the same run in SimuLife (blue cells are necrotic)

The values of the parameters used in the model can be changed easily. Playing with the values of these parameters, as well as with other parts of the model, helped to gain a better understanding of their effect on the behavior of the system.

### High vs. low oxygen secretion

**Fig. 10:**
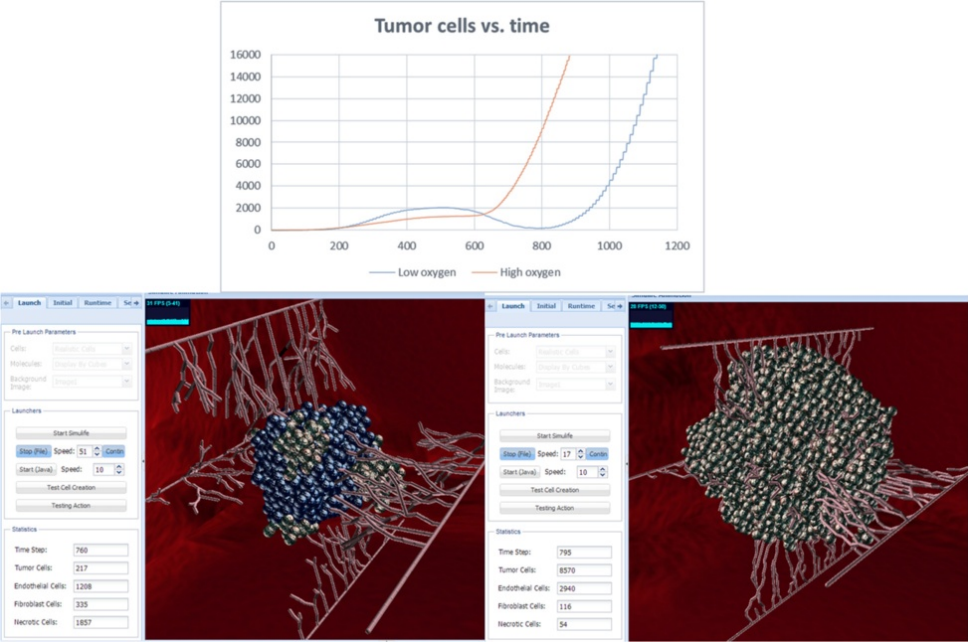
High vs. low oxygen secretion simulations. Top: graph of tumor cells showing that at a low oxygen level the tumor cells drop to almost zero at ~800ts but recover and reach 16,000 cells at ~1100ts, whereas at a high oxygen level they do not demonstrate a drop and reach 16,000 cells shortly after ~800ts. Bottom: images of these runs in SimuLife (left for low oxygen, right for high oxygen), presenting amounts on their left tab. Both images are presented at approximately the same time step – at low oxygen only few active tumor cells are present, at high oxygen the tumor consists of many cells where almost all are active

Playing with the parameter *OxygenSecretionAmount*, which represents the number of oxygen units secreted every pulse in the simulation, reveals that very low oxygen secretion slows down the growth of the tumor until it is almost completely eliminated. However, as long as some cells are still active, they manage to recover from the situation and end up growing into a large tumor, as large as when *OxygenSecretionAmount* is high, but later in time (Fig. 10). Therefore, above a certain threshold of minimum oxygen secretion, the tumor and vessels will eventually develop to their full competence. Below this threshold, a tumor will not develop in the model. Although in reality tumor cells deprived of oxygen can produce energy by fermenting sugars (Warburg effect, [80]), and become even more aggressive, the oxygen in our model represents both actual oxygen and nutrients.

### High vs. low VEGF secretion

**Fig. 11:**
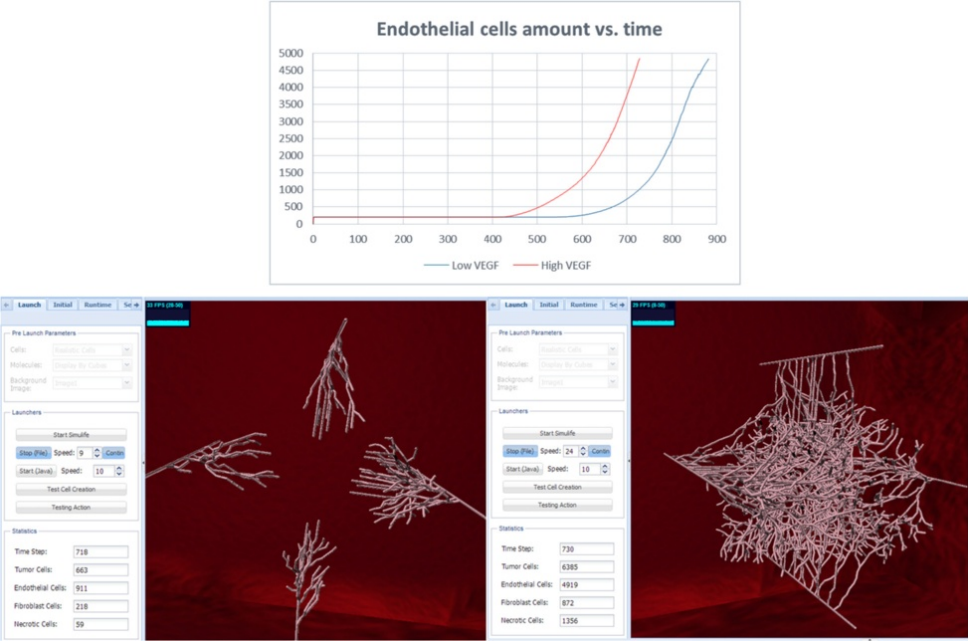
High vs. low VEGF secretion simulations. Top: graph of endothelial cells showing that at low VEGF the endothelial cells are activated at ~600ts and reach ~5000 cells at ~900ts, much later than at high VEGF secretion, which begins at ~400ts and reaches ~5000 cells at ~700ts. Bottom: images of these runs in SimuLife (left for low VEGF, right for high VEGF), presenting amounts on their left tab. Both images are presented at approximately the same time step–at low VEGF angiogenesis has only begun, whereas at high VEGF there are many activated and branched vessels

VEGF secretion by the tumor cells is what recruits the vessels towards the tumor in order to supply it with oxygen/nutrients. Playing with the parameter *VEGFSecretionAmount*, the amount of VEGF units secreted by the tumor cells at every pulse in the simulation, reveals that, here too, very low VEGF secretion results in tumor and vessels similar to those resulting from high VEGF, although it takes longer, as vessels take more time to become activated by the VEGF (Fig. 11). This indicates that the tumor system is insensitive to the amount of VEGF, as long as there is some VEGF secreted by the tumor cells. Since there are many secreting cells in a tumor, the VEGF will eventually reach and activate the nearby vessels.

### High vs. low angiogenic switch threshold

**Fig. 12:**
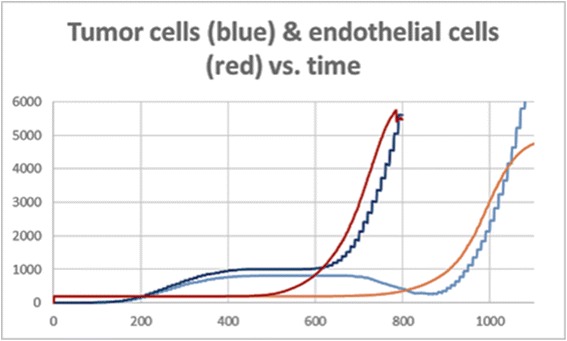
High vs. low angiogenic switch threshold simulations. Graph of tumor cells and endothelial cells at low angiogenic switch threshold (light colors), and high angiogenic switch thresholds (dark colors). They reach approximately the same levels, but at a time shift of ~200ts. Also, at high angiogenic thresholds we see that the tumor almost dies at ~900ts, but then recovers

In order for the endothelial cell to be activated for angiogenesis, it needs to meet a minimum amount of VEGF in a certain amount of time [76]. Playing with the parameter *AngiogenicSwitchThreshold*, which represents the minimum amount of VEGF needed to initiate angiogenesis, reveals once again that there is a threshold for this parameter; any value above it means that it will take the vessels too long to become activated, and they won’t arrive at the tumor in time before it dies. Below this value the tumor survives and arrives at the same end point, but the time it takes it takes the tumor to develop depends on the value: the lower the threshold the faster the occurrence of angiogenesis (Fig. 12).

### High vs. low hypoxia level

Hypoxia (insufficient oxygen supply) in the model occurs after a cell has not consumed a sufficient amount of oxygen for the duration of *HypoxiaLevel* time steps. At this point the cell continues to live but cannot proliferate. If it is a tumor cell, it also begins to secrete VEGF in order to recruit blood vessels. At high levels of *HypoxiaLevel* the tumor grows more before needing the help of angiogenesis. When the tumor cells do eventually secrete VEGF molecules, there are many cells that do so, hence angiogenesis occurs fast and the tumor continues to grow. When *HypoxiaLevel* is too low, the tumor cells lose their ability to proliferate very fast and so the VEGF they secrete is not enough to recruit the vessels in time before the tumor dies. Once again, values higher than the minimum threshold will eventually result in a developed tumor, but at different time durations–the lower the value the more time it will take.

### High vs. low anoxia level

Anoxia (total depletion in the level of oxygen) refers to when the cell is completely out of oxygen and cannot continue to live. At this stage it will become necrotic (an un-programmed, unnaturally occurring cell death–as opposed to apoptosis). This occurs at a later stage than hypoxia, after the cell has had insufficient oxygen for a duration of *AnoxiaLevel* time steps. When this level is very high no necrotic core occurs, since although all cells may not be proliferating, they live on. At very low levels the entire tumor dies out very fast. Values in between form necrotic cores of different sizes, depending on the value of the parameter, but since the outer cells continue to receive oxygen, the tumor as a whole continues to develop.

A list of the VEGF and Oxygen parameters that were used for analysis, as well as the range of values thereof that ensures tumor recovery and development can be found in Additional file 1.

In each of the conditions described above, if the tumor recovered from just a few surviving cells, those cells are considered the stronger, more aggressive ones (survival of the fittest [81]).

This analysis indicates that the tumor is in fact a very robust system. On the one hand, each of the key parameters has a threshold, which, if crossed, the tumor does not develop. However, at the same time, any value within the range allows the tumor to eventually become fully developed, even if it takes longer and the tumor has to overcome tough conditions. (A short clip showing these results in SimuLife can be found in Additional file 2.) The issue of robustness of the tumor was raised in the past [82], where it was suggested that this fact calls for new therapeutics.

Additional file 2 Video clip of the cancer model in SimuLife. A recording of the process of the model from Statecharts to the SimuLife animation presenting some of the biological results.

**Fig. 13:**
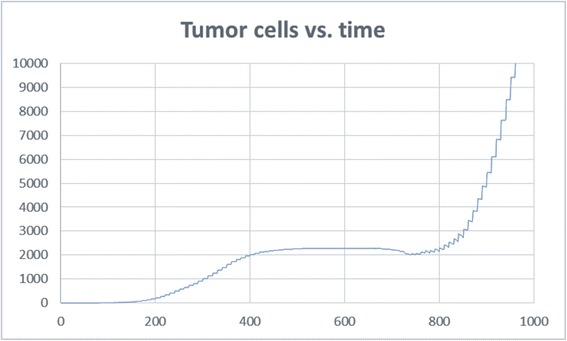
Tumor cell dynamics. Just before 800ts there is a turning point for the tumor and a phase transition

Moreover, what we saw in all runs of the model, especially in those where the tumor recovered from tough conditions, was the phase transition in the tumor cell dynamics. At first, the tumor grows linearly, consuming oxygen from its surroundings. The growth then slows down, and sometimes even decreases, due to low oxygen. When enough blood vessels finally arrive at the tumor and feed it with a large quantity of oxygen, the tumor cells start growing exponentially (Fig. 13). At this point, the amount of cells increases, requiring more oxygen, and the cells therefore secrete more VEGF, and in turn receive more oxygen. In this way, the tumor and its surroundings maintain themselves. This is a critical turning point for the tumor, since if it does not manage to pass this point, it will simply die. This finding supports the need to treat tumors as early as possible [83, 84].

## Conclusions

Cancer affects many people. It is researched in numerous labs around the world, in order to understand its function and behavior better and to develop possible treatments. Cancer is a multi-scale and complex system, where the tumor and its microenvironment work together in a way similar to an organ. Applying advanced computational techniques to this system is complementary to classical ongoing research. It enables the integration of a variety of data and helps one see the big picture.

In our research we used the language of Statecharts with the Rhapsody tool in order to create a comprehensive 3D model of a cancerous solid tumor, together with its microenvironment. The model was constructed in a bottom up method, where the behavior of each kind of participating component was built individually, and the system’s overall dynamic behavior emerged from those of the elements thereof.

In order to better understand biological models there is a need for detailed and realistic visualization. For this purpose the SimuLife tool was developed, where an animation is dynamically constructed at real time, producing an interactive visualization of the system’s runs. It shows the tumor cells at their precise 3D locations, together with the blood vessels that consist of the individual endothelial cells. The blood vessels elongate towards the tumor by following the VEGF gradient, and in turn secrete oxygen. Cell proliferation or death (in the case of tumor cells this is necrosis), and molecule movement can be observed too. SimuLife allows one to easily play with the animation, send commands back to the model during runtime and observe the immediate resulting output. A more detailed description of SimuLife and its abilities can be found in [50].

Using the SimuLife tool, we were able to see that the model matched the behavior of a solid tumor in a number of ways: It developed an inner necrotic core, the branching of the blood vessels occurred more often as they approached the tumor, the VEGF molecules that were secreted from the tumor cells diffused and were distributed throughout space until reaching the blood vessels, the oxygen secreted from the endothelial cells was also diffused and finally reached and was consumed by the tumor cells. In addition, the dynamic behavior of the various cells showed patterns similar to those found in real tumors. Further verification was carried out to test known cases within the model. This included verifying that the tumor cannot continue to grow without angiogenesis, that a non-cancerous cell can continue to live on without angiogenesis, and that when placing blood vessels far from the tumor angiogenesis does not take place.

From analyzing the components of the model by looking at their qualitative and quantitative dynamics, playing with the different parameters, and inspecting and analyzing the resulting animations, we conclude that the tumor has a turning point, which depends on thresholds of the key parameters that effect amounts of VEGF and oxygen. At this point, the tumor either dies, or recovers and continues to develop to become a full, actively growing tumor. Thus, the tumor’s growth may be halted or declined while it waits for the blood vessels to deliver oxygen. If the oxygen arrives in time, while there are still surviving tumor cells, a phase transition will occur in the tumor’s growth, and from a linear growth rate it will suddenly start growing exponentially. The VEGF and oxygen parameters (amount secreted, consumption, activation threshold….) are what affect the fate of the tumor in this case. If the thresholds are met and the fate of the tumor is to continue, the values of these parameters do not make a big difference. A tumor that survives will develop similarly within the range of allowed values, the main difference being in the time it takes.

It is known that a tumor cannot continue to grow above a certain size (~1 mm^3^) without angiogenesis [6, 85, 86]. The angiogenic switch point is when the blood vessels have bound enough VEGF to begin angiogenesis [87] and deliver oxygen and nutrients to the tumor. Evidence has shown that tumor growth is slow and linear before vascularization and rapid and nearly exponential after vascularization [88, 89]. Therefore, the essential need of VEGF to recruit blood vessels, and oxygen and nutrients for the tumor to continue growing is confirmed. Here, we suggest that not only is there an angiogenic switch turning point that causes the tumor to enter exponential growth, but there is also a recovery turning point. This means that: 1) if the tumor cells secrete VEGF in any amount, the blood vessels will eventually arrive at the tumor, and 2) if at least some active tumor cells are alive when the blood vessels arrive at the tumor, and if they deliver a sufficient amount of oxygen for those cells to continue their activity, the tumor will eventually become fully developed, even if it takes longer. This also suggests that the tumor, together with its microenvironment, is a robust system, reaching its maximum outcome if the minimum conditions are met, regardless of the actual amounts. It therefore seems like the tumor does not economize resources, and sends well over the needed angiogenic factors.

These conclusions may provide further evidence as to why inhibiting VEGF, or reducing the supply of oxygen and nutrients to the tumor, does not always result in its complete elimination. This is especially relevant to anti-cancer treatments other than chemotherapy, such as VEGF inhibitors [90], or destruction of the tumor’s surrounding blood vessels and hence elimination of its oxygen supply, such as is done in photodynamic therapy [91–95]. These treatments, according to the model’s results, can extend the time it takes the tumor to become fully developed.

## Methods

### The Statecharts modeling language

Our model was designed using the visual language of Statecharts [96, 97], which was invented as a system engineering tool to aid in the design of complex reactive systems [96, 98], where the components react to each other and to the environment. The language makes possible the dynamic and visual specification and execution of reactive behavior via the use of intuitive, yet completely formal and fully executable, diagrams. Statecharts describe discrete behavior using states, and events that cause transitions between the states. Orthogonal/concurrent states may also be specified, such that the system or parts thereof may be in several different states simultaneously, in accordance with the different stages of the simulation. In addition, the language is hierarchical, so that states may contain substates, which enable description at multiple levels, as well as level-rich transitions. The object-oriented version of the Statecharts language [97] is based on an *intra*-object philosophy; i.e., on supplying the full description of the internal behavior of each of the participating objects.

Statecharts and their execution are supported by several appropriate tools, such as Rhapsody, which was co-designed by the second-listed author, and is available from IBM (www.ibm.com/software/awdtools/rhapsody/).

The language has become very useful in modeling a variety of biological systems (see past [44–47, 99]), since these are in fact complex reactive systems; they interact with, and respond to, both the environment and other parts within the system [48, 98]. We have used Statecharts to specify the behavior of the individual entities that take part in the cancer process, in order to capture the dynamic behavior and morphology of the system.

### SimuLife, a 3D animation tool

SimuLife is an interactive animation tool we have built for visualizing models of cellular biology [50]. It can receive inputs from the model, as well as send information to the model (both sent as XML files) and draws the appropriate animated graphics based on the changes. This makes it possible to see the ongoing events of the entire model on the screen in real time [51, 52]. SimuLife is based on WebGL (Web Graphics Library), a JavaScript API (THREE.js framework in our case) for rendering interactive 3D graphics within any compatible web browser without the use of plug-ins. The client side is Chrome and the communication with external engines is done via sockets. The realistic images used within SimuLife (e.g., of a cell) are in the COLLADA format.

## Additional files

Additional file 1. Further details of the model. Details regarding the specification of the model, together with a list of the parameters used in the model and their default values, and the range of values for VEGF and oxygen parameters that ensure tumor recovery. (PDF 244 kb)

## Abbreviations

CAF: Cancer associated fibroblast
ECM: Extra cellular matrix
FGF: Fibroblast growth factor
HGF: Hepatocyte growth factor
RA: Reactive animation
VEGF: Vascular endothelial growth factor
