# Supplementary material for: The tumor as an organ: comprehensive spatial and temporal modeling of the tumor and its microenvironment
Source: BMC Bioinformatics. 2016 Aug 24;17(1):317. doi: 10.1186/s12859-016-1168-5 (PMC4995621; doi:10.1186/s12859-016-1168-5)
Supplement: Additional file 1. — Further details of the model. Details regarding the specification of the model, together with a list of the parameters used in the model and their default values, and the range of values for VEGF and oxygen parameters that ensure tumor recovery. (PDF 244 kb) [file 12859_2016_1168_MOESM1_ESM.pdf]

Additional file 1:

The tumor as an organ

Comprehensive spatial and temporal modeling of the tumor and its microenvironment

Naamah Bloch and David Harel
Dept. of Computer Science and Applied Mathematics, Weizmann Institute of Science, Rehovot, Israel

Cancer model specification and behavior

In the model, each of the entities is represented as a reactive object that behaves according to its current state and environment. The behavior of each object is given as a statechart. The tumor cell, endothelial cell, and the fibroblast cell, all inherit from the *Cell* statechart.

The *Cell* statechart consists of four orthogonal states: (1) *EnvSense*, which constantly senses the environment around it for presence of molecules, and, in the case of detection of some possible input, signals the *Input* state to become activated; (2) *Input,* which carries out the actual uptake of the molecules from the immediate surroundings of the cell, while calculating their levels; (3) *Output*, which controls the secretion of relevant molecules to the environment when needed; (4) *StateIn*, which describes the current state of the cell – whether *Naïve*, *Activated*, or *Dead*. The *Activated* state contains a sub-chart that describes if the cell is proliferating or not, and for the endothelial cell also defines the branching process.

The *Area* object is the controller of the system. It describes the overall "world" of the system and handles the three dimensional lattice of the objects' positions within the world, the molecules' positions, and the time steps of the system, as well as taking care of outputting the data to files. It contains many functions that are used throughout the simulation.

Each of the objects is also linked to a *Position* entity, which holds their specific and current x, y, z coordinates.

When the model is executed, the dynamics of the system emerge as a product of the behavior of all the entities and the interactions between them.

Cell size

In order to create a realistic tumor, there was a need to properly define the position occupied by a cell. If it were to take up a single position the tumor would take on a symmetric form. To overcome this problem, we defined the resolution of the cell to be a cube of any edge length in terms of the number of positions, where if it is above 1, the tumor will take on a non-symmetric form. This is due to the fact that a neighboring cell does not necessarily have to be an exact position above or below it, but can also be half a cell-size above (in the case of cell resolution of 2), etc.

Tumor cell survival & proliferation

The tumor cells can only proliferate under the condition that there is free space, the size of a cell, around them. The position of the new cell is chosen randomly from any

adjacent place around the cell (including the diagonals). A tumor cell constantly consumes oxygen and will proliferate only if it has a sufficient amount of oxygen. The cell consumes the oxygen it needs randomly from the cell and its immediate surroundings of one position in any direction around it. If it does not have a sufficient amount, it will become hypoxic (deprivation of adequate oxygen supply) and stop proliferating. In parallel it will start sending angiogenic factors, VEGF. If it continues to lack sufficient amounts of oxygen it will become anoxic (extreme decrease in the level of oxygen) and eventually die (become necrotic). An active hypoxic cell that returns to consume enough oxygen will stop secreting VEGF.

VEGF diffusion

VEGF diffusion is a continuous process, but we chose to represent it in its most basic form – as individual molecules that move in a random walk fashion. Each molecule that is secreted takes on the initial location of the tumor cell from which it came, and takes up exactly one position. It can move into any adjacent position (including all diagonals), even if that position is taken up by another molecule or cell. Each VEGF object can also represent more than a single molecule. While implementing this, there was a need to check if the collection of the individual molecules moving randomly indeed creates the overall diffusion phenomenon. This involved testing the amount of molecules that each cell secretes, the rate of secretion, and the rate of the molecules' random walk, and also deciding on what happens to molecules that reach the edge; should they disappear, come back, or stay at the edge? These tests resulted in a successful diffusion progression.

Every VEGF molecule in the model represents ~10 real VEGF molecules:

In [1] VEGF values in tumor were measured. From table 6 in this paper, the total tumor VEGF that was measured was 13 pg/mg tumor. This value was converted to 0.3 pmol/cm^3 tissue to be used in their model. Since the world of our model is of size 100*100*100 positions, where each position represents 5 μm, it is 500^3 μm^3 = 0.05^3 cm^3 = 0.000125 cm^3. Therefore there will be 0.3*0.000125 pmol in our model's world = 0.0000375pmol = 3.75 *10^{-5}*10^{-12}mol = 3.75*10^{-17}*6 *10^{23}mol = 22.5 million molecules. In our model, at the stage when the tumor is developed and vascularized the average number of VEGF molecules is 2-3 million, meaning that each molecule in the model represents ~10 real VEGF molecules. This is a reasonable number, allowing, on the one hand to represent only a fraction of the real number, but only to one order of magnitude, meaning that it is sensitive enough to observe changes that occur to the molecules.

Oxygen

Oxygen, like VEGF, is treated as individual molecules that move in a random walk fashion. It is secreted by every endothelial cell at a constant rate, and takes on the initial location of the endothelial cell that secreted it. In the initiation of the simulation there is a constant amount of oxygen in each position in order to mimic the constant oxygen levels in the body, and to allow time for the oxygen to diffuse from the blood vessels. Here too, each molecule takes up one position and can move into any adjacent position (including diagonals), even if that position is taken up by another molecule or cell.

Every oxygen molecule in the model represents ~7 million real oxygen molecules:

In [2], 10mmHg was found to be the median partial pressure of O_2 in tumors. By using the ideal gas equation of PV=nRt, where P=pressure in pascals = 10mmHg*133 = 1333pascals, V=volume in cubic meteres, in our model, the world is 500^3 μm^3 = 0.0005^3 m^3 = 0.000000000125 m^3 = $1.25*10^{-10}$ m^3, R (constant) =8.314, T=temperature in kelvin = 37 degrees Celsius in the body = 310 Kelvin. Therefore n (number of mols) = $1333*1.25*10^{-10}/ (8.314*310)$ = $6.46*10^{-11}$mols = $6.46*10^{-11}*6*10^{23}$=~$39*10^{12}$ oxygen molecules in the system. This corresponds to ~5-6 million molecules on average in the model, meaning that each oxygen molecule in the model represents ~7 million molecules. This too is a reasonable number, as there are many more oxygen molecules, as they are much smaller and are largely consumed by all cells everywhere all the time for survival. It is obviously impossible to represent them individually and their gradient is less important than with the VEGF molecule, but more importantly is to be able to observe where enough oxygen is present.

Molecule elimination

Both VEGF and oxygen that pass the edge of the world or are consumed by the appropriate cells, are eliminated from the system.

Angiogenesis

The angiogenesis process in the model occurs along the following steps:

- Endothelial cell checks for VEGF around it (environment sensing).
- If VEGF is found it binds to the cell according to a binding probability, and the molecule is eliminated.

- If the levels of VEGF molecules that bind to the cell pass a certain threshold (the angiogenic switch), the cell becomes activated and begins the process of angiogenesis.
- This process is guided by the VEGF gradient that the cell senses at each point.

Angiogenic switch

In order for the endothelial cell to be activated for angiogenesis it needs to pass the threshold of meeting a certain amount of VEGF's in a certain amount of time [3].

Endothelial proliferation

The blood vessels in the model are represented by individual endothelial cells arranged linearly. This representation was chosen in order to continue the trend of modeling basic entities, although it could have also been modeled by representing portions of the vessel. In biology, during angiogenesis, the cell that is activated to begin angiogenesis becomes the lead cell (the tip) that guides the migration. The proliferation of the endothelial cells occurs at the stem of the vessel and allows for elongation of the cells in front of it. In the model, this was implemented somewhat differently; the proliferation of the endothelial cells occurs at the tip and this elongates the vessel.

Endothelial direction

The direction taken by the endothelial cells during angiogenesis has a great effect on the final spatial organization of the vessels. In our model, the cell scans a vector radius around it and calculates the geometric averages of the amount of VEGFs and moves in the direction of the highest average. The radius scanned can be increased or

decreased to change the manner of the vessel's elongation; the larger the radius being scanned the more directly the vessel will progress towards the tumor, and the smaller the radius the more twists and turns it will have while progressing towards the tumor in an indirect manner.

Endothelial elongation

If the endothelial cell is directed by the VEGF gradient to elongate into a position occupied by another endothelial cell, it will join this vessel and stop elongating. If, however, the position is occupied by a tumor cell, the endothelial cell will try to find an empty space in the closest possible direction, so as to continue its elongation. In addition any endothelial cell that is a part of an angiogenic vessel can also split and branch out of its main vessel if it binds to a high amount of VEGF in a specified time. The continuation of the endothelial cell progression depends on the presence of VEGF molecules. Without a sufficient amount thereof it will not continue to proliferate and the vessel will halt the elongation.

Delta-Notch inhibition

Delta-notch lateral inhibition signaling is involved in cell-to-cell communication and regulates the determination of various cell fates. Among cells that have the potential to adopt the same fate, lateral inhibition specifies some cells for a primary or preferred fate and others for a secondary or alternative fate [4, 5]. In the case of endothelial cells, the activated cell inhibits its adjacent cells, thus preventing them from becoming active.

List of parameters

Table 1: A list of the parameters used in the cancer model.

Parameter	Description	Default value
Hypoxia Level	Number of time steps that the cell does not have sufficient oxygen and starts secreting VEGF	30
Anoxia Level	Number of time steps that the cell does not have sufficient oxygen and dies	200
Oxygen Consumption	The amount of oxygen a cell consumes every time step	16
Cell Size	The number of pixles to the power of 3 that each cell occupies	2
VEGF Secretion Amount	Number of VEGF’s secreted in each pulse	10
FGF Secretion Amount	Number of FGF’s secreted in each pulse	100
Duration For Summing HGF’s	The number of time steps back that sum up HGF amount	8
Angiogenic switch threshold	Minimum VEGF level to initiate angiogenesis	13
Endothelial sensing radius	The radius that the cell checks around it for VEGF’s	3
Min VEGF for survival	The minimum amount of VEGF needed for survival of the endothelial cell	2
Oxygen Secretion Amount	Number of oxygen units secreted in each pulse	100
VEGF Summing Duration	The number of time steps back that sum up VEGF amount	8
Initial Vessels Growth	The way in which the initial vessels will grow	1
Duration for summing FGF’s	The number of time steps back that sum up FGF amount	8
Amount HGF secreted	Number of HGF’s secreted in each pulse	10
Fibroblast sensing radius	The radius that the cell checks around it for tumor cells	3
Probability to become a CAF	The probability for a normal fibroblast to become a CAF (cancer associated fibroblast)	0.02
Model World Size	Pixles to the power of 3 that determine the full size of the world modeled	100
Initial oxygen level	The initial amount of oxygen in each pixel	10
Initial vessels	The number of initial vessels	4
Oxygen moving distance	The number of pixles the oxygen will move every step	1
Proliferation duration	Time it takes for cell to proliferate	10
VEGF moving distance	The number of pixles the VEGF will move every step	2

FGF moving distance	The number of pixles the FGF will move every step	1
HGF moving distance	The number of pixles the HGF will move every step	1
Oxygen Move Rate	The time in which the oxygen makes one move	1
VEGF Move Rate	The time in which the VEGF makes one move	2
FGF Move Rate	The time in which the FGF makes one move	2
HGF Move Rate	The time in which the HGF makes one move	2
Secretion Rate	The time for one pulse of VEGF/oxygen to be secreted	10
Number of initial fibroblasts	Number of initial fibroblasts	100
Probability to become cancerous	Initial probability for a normal cell to become cancerous	0.2
Probability to proliferate	Initial probability for a cell to proliferate at a given moment	0.6
Probability to bind	Initial probability for a cell to bind a molecule	0.8
Simulation time	Duration of simulation run	1500

A list of the parameters used in the cancer model together with the description of each parameter and its default value. The default values are used to achieve a standard run and can change according to changes in other parameters, as well as due to further development of the model. Parameters are unitless but are quantified relative to each other. Each time step (ts) in the model is approximately equal to 1-2 hours, and each 10^3 cells in the model are approximately 100^3 actual cells.

Table 2: Range of values for VEGF and oxygen parameters that ensure tumor recovery

**Parameter**	**Value range for tumor recovery**
Oxygen secretion amount	>4
VEGF secretion amount	>0
Angiogenic switch threshold	<100
Hypoxia level	>15
Anoxia level	>100

List of the VEGF and Oxygen parameters that were used for analysis, indicating the range of values for each of them that ensures tumor recovery and development, as long as all other parameters in the model are set to their default values.

References

1. Stefanini MO, Wu FT, Mac Gabhann F, Popel AS: **A compartment model of VEGF distribution in blood, healthy and diseased tissues**. *BMC systems biology* 2008, **2**:77.
2. Hockel M, Vaupel P: **Tumor hypoxia: definitions and current clinical, biologic, and molecular aspects**. *Journal of the National Cancer Institute* 2001, **93**(4):266-276.
3. Ribatti D, Nico B, Crivellato E, Roccaro AM, Vacca A: **The history of the angiogenic switch concept**. *Leukemia : official journal of the Leukemia Society of America, Leukemia Research Fund, UK* 2007, **21**(1):44-52.
4. Appel B, Givan LA, Eisen JS: **Delta-Notch signaling and lateral inhibition in zebrafish spinal cord development**. *BMC developmental biology* 2001, **1**:13.
5. Harvey NL: **To sprout or "Notch" to sprout?** *Blood* 2011, **118**(4):836-837.
